# Supramolecular Chemistry: Host–Guest Molecular Complexes

**DOI:** 10.3390/molecules26133995

**Published:** 2021-06-30

**Authors:** Sadaf Bashir Khan, Shern-Long Lee

**Affiliations:** 1 Institute for Advanced Study, Shenzhen University, Shenzhen 518060, China; 2 Laboratory of Optoelectronic Devices and Systems of Ministry of Education and Guangdong Province, College of Optoelectronic Engineering, Shenzhen University, Shenzhen 518060, China

**Keywords:** scanning tunneling microscopy (STM), interaction, two-dimensional (2D), porous, guest molecules, host–guest (HG)

## Abstract

In recent times, researchers have emphasized practical approaches for capturing coordinated and selective guest entrap. The physisorbed nanoporous supramolecular complexes have been widely used to restrain various guest species on compact supporting surfaces. The host–guest (HG) interactions in two-dimensional (2D) permeable porous linkages are growing expeditiously due to their future applications in biocatalysis, separation technology, or nanoscale patterning. The different crystal-like nanoporous network has been acquired to enclose and trap guest molecules of various dimensions and contours. The host centers have been lumped together via noncovalent interactions (such as hydrogen bonds, van der Waals (vdW) interactions, or coordinate bonds). In this review article, we enlighten and elucidate recent progress in HG chemistry, explored via scanning tunneling microscopy (STM). We summarize the synthesis, design, and characterization of typical HG structural design examined on various substrates, under ambient surroundings at the liquid-solid (LS) interface, or during ultrahigh vacuum (UHV). We emphasize isoreticular complexes, vibrant HG coordination, or hosts functional cavities responsive to the applied stimulus. Finally, we critically discuss the significant challenges in advancing this developing electrochemical field.

## 1. Introduction

In host–guest (HG) interaction, distinctive structural complexes development occurs via non-covalent associations [1]. There is a growing curiosity in executing supramolecular HG structures for assembling organic solvents and aqueous solutions on compact planes [2]. Previous studies reveal that compact, dense planes offer an adequate degree of crystallinity in host linkage, favoring efficient guest traps. It also delivers added firmness and constancy to the subsequent HG supramolecular assemblies using molecule substrate interactions [3,4]. Often, substrate accumulated host complexes show particularity in guest molecules displaying crystalline configurations analogous to enzymes or zeolites [4,5]. These nanostructured host planes can be easily assimilated into realistic purposeful supramolecular structures leading towards prospective applications in molecular sensors, drug delivery, flat panel display devices, cosmetics, catalysis, or separation equipment [6]. HG chemistry is investigated either in solution or on solid surfaces, each having its unique physiognomies and molecular recognition [7,8,9,10]. The conjointly specific interactions among the host system and the guest links take place over atomic dimensions [11,12]. At sub-molecular resolution, STM [11,13] helps to spot these HG links on the condition that it proceeds on the atomically plane conductive smooth substrate [14,15,16]. Molecular identification in traditional solution-phase host–guest interaction is more often comprehended using indirect methods, including calorimetry technique to measure the degree of heat alteration, UV-vis absorption, or chemical shift variation. The experimental evidence gathered from these measurements offers statistics on the selectivity and strength of intermolecular interactions, letting researchers evaluating step-by-step procedures keep the different influencing parameters consistent throughout the process. Even though these procedures are exceedingly progressing and scientifically demanding, they cannot yet capture the direct visual structure of the HG multiplexes. In this regard, STM offers fundamental facts about HG centers; additionally, it permits monitoring the dynamics and active features of this coordination, hence apprehending molecular proceedings. STM has advanced as a multipurpose method for investigating supramolecular HG networks operative in various environmental conditions [17,18]. On a flat conductive surface, the HG complex is every so often acquired through molecular self-accumulation.

The carbon-based molecules accumulate in self-assemblies to generate a host network. The host complexes encompass cavities in the form of nanowells comprising of a single molecule. In these nanocavities, the guest molecules can adsorb or trap within these networks. Naturally, these HG linkage systems show persistence via vdW interactions, hydrogen bonding, metal-ligand association, or halogen bonding. At the LS interface, dynamic co-adsorption of solvent molecules takes place to stabilize the generated host complexes. Guests’ molecules become restrained and immobilized when the dimensions, including magnitude, feature, and contour of guest molecules, are suitable with the cavities offered by the host network. The constraint on guest molecules arises depending on solvent desorption. The guest molecules usually possess higher adsorption energy in comparison to solvent molecules. The attractive dispersion or diffusion plays a significant role in maintaining the equilibrium and stabilization among guest molecules and host networks, including the underlying surface. As a result, the HG surface interactions are often surface-dependent because both the host and guest molecules can be transported on the surface simultaneously. A complex and dynamic environment is often observed during liquid-solid (LS) solution interactions compared to UHV surroundings because of molecule-solvent and solvent-surface interactions. Besides this, added molecular and intermolecular surface interactions were present in the self-assemblies. Moreover, LS interactions deliver encouraging environments for molecular dynamics so that guest trapping occurs near equilibrium. However, the supporting substrate selection is narrow to the ones that possess stability in ambient conditions and do not experience oxidation. Commonly, the more frequently used substrates were highly oriented pyrolytic graphite (HOPG) or Au (111). However, MoS_2_ is also used to support STM experimentations in the ambient environment [19].

Even though the LS interface offers an ‘apparent life’ sight of assembly development, ultra-high vacuum experimentations require special care, such as the ultra-clean environment. In UHV, the molecular assembly was carried out utilizing the organic molecular beam epitaxy (OMBE) system permitting precise mechanism over atomic ratios and stratum width. Earlier, under UHV conditions, various crystal surfaces comprising of metals such as gold, copper, platinum, or silver were used as supporting surfaces for the study of HG chemistry [20]. In UHV, the self-assembly formation takes place in a vacuum, so the surface temperature shows consistency. Moreover, it can be easily controlled, which allows for optimized annealing and even viewing at low temperatures. The surface is an essential feature in HG interfaces as it fundamentally directs the freedom of movement among molecules due to adsorption, self-healing, or self-alignment. During UHV settings, high-temperature annealing persuades the dynamics in supramolecular hybrids. At the LS interface, the annealing is sometimes avoided due to solvent evaporation. It is a significant issue in metals because metals intermingle strongly with aromatic molecular structures. As a result, to attain and monitor self-accumulated (long-range) complexes of physisorbed molecules is tricky on metallic substrates compared to HOPG because of the greater diffusion obstructions. That is the reason HOPG offers a wide range of multicomponent self-assemblies or supramolecular hybrids studies without influencing the molecular dynamics. Numerous correspondences subsist between HG approaches active on surfaces and those implemented in solution. STM plays an influential role in investigating and analyzing the intrinsically porous network encompassing stable covalent cavities and extrinsic permeable host complexes. The inherent pore in an isolated molecule exists due to molecule configuration. The intrinsically porous host system comprises macrocyclic compounds such as cyclodextrins [21], crown ethers [22], and calixarenes [23]. In comparison, the extrinsic porosity is an after-effect generated by constituent molecules due to covalent or non-covalent assemblage. Earlier different research groups focus on extrinsically porous structures that self-assemble upon adsorption supported on solid planes to generate a host system. Sometimes, the host establishing molecules act as guests, known as auto host-guest systems [24]. Usually, the HG network assembly comprises two components. The advanced order multi-component systems involve four different molecular components in which more than one kind of guest particle was accumulated in a paternal host system [25]. Thus, it is essential to know that the HG system comprises a multi-component system; still, the multicomponent network did not represent HG coordination.

In the present review, we deliver a detailed description of substrate sustained HG interaction advancement via emphasizing prominent illustrations from previous literature. The present review is sectioned into the following subdivisions, as mentioned below. After concisely introducing the revolutionary patterns, we comprehensively define well-organized host linkages displaying isoreticular surfaces having accessible voids. Next, innovative HG structures, including supramolecular organic frameworks (SOFs) [26] and covalent organic frameworks [27] (COFs), were discussed. The succeeding part of the review includes the analysis of numerous characteristics of HG interaction, comprising the multi-component energetic network, selection of guest integration, incentive receptive coordination, and field-induced system. In the concluding part, we provide a summary.

## 2. The Development of the HG Network

**Figure 1:**
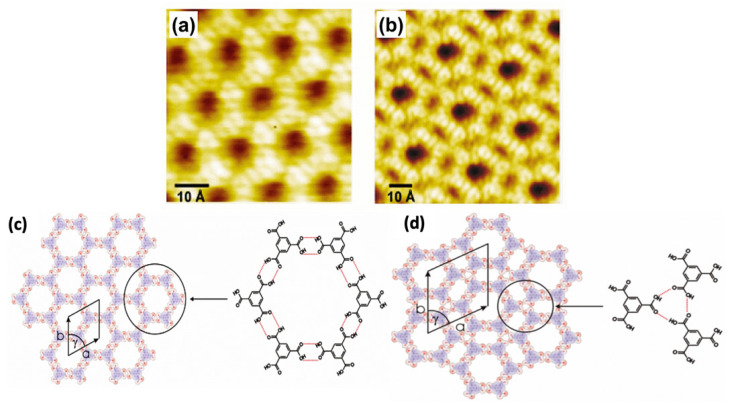
STM image of TMA on graphite. (**a**) Chicken-wire and (**b**) flower assembly (−1.4 V, I = 126 pA) Schematic diagram of the (**c**) chicken-wire structure (a = b = 17.2 ± 1 Å, Υ = 60°), (**d**) flower structure (a = b = 27.0 ± 1 Å, Υ = 60°) Reproduced from ref. [1] with permission from Wiley-VCH Verlag GmbH & Co. KGaA.

In the early 2000s, single molecules having two-dimensional (2D) porous morphology were witnessed. The initial example is the innovative description of trimesic acid; TMA (1,3,5-tricarboxylic acid), which is a hydrogen-bonded porous hexagonal system generated via benzene on HOPG [1]. TMA yields a cyclic hexamer through resonance stabilization via a hydrogen attachment. Two different polymorphs of TMA were observed under the UHV environment, i.e., 1. Flower assemblies, 2. Chicken wire (honeycomb), as shown in Figure 1a,b [1]. The flower assembly comprises comparatively denser assemblies of TMA molecules in comparison to the chicken wire assembly. A hexagonal lattice was displayed in both assemblies. The TMA molecules display a rim in a sporadically organized manner, having an interior diameter (D) of ~1.1 nm. These hydrogen-bonded systems were exceptionally adaptable and could be easily invented in UHV surroundings or at the LS interface on several planes [22,28]. TMA is considered a unique and durable self-accumulated host system. TMA immobilizes different guest molecules depending on the dimensions, contour, and size, such as coronene (C_24_H_12_) [29,30]. C_24_H_12_ is a D_6_h symmetric polycyclic compound encompassing one central and six adjacent C_6_ rings. Coronene maps onto the graphite/graphene basal plane and can be used as a finite model for the carbon allotropes, having 2D extended assemblies. According to electrochemical investigations, coronene can accept up to two electrons heterocirculenes [31] and C_60_ [28]. However, it is slightly difficult to predict the composite crystal structure and associated interactions between interlinked molecules and their crystal symmetry in crystal engineering. Recently Xiao, W.D., and his research fellows verified the impact of molecular symmetry and crystallization of 2D heterocirculenes on the Au (111) substrate via STM observing D_8_h symmetric sulflower and D_4_h symmetric selenosulflower coverage from submonolayer to monolayer [32].

Earlier literature demonstrates that TMA paved the basis of host setups established on directional hydrogen bonding between carboxylic groups. The preliminary investigations on substrate restrained interface interactions demonstrate a complicated bicomponent host system. In the TMA molecule, chicken wire or flower phase co-occur with various proportions depending on the synthesis parameters. A single TMA molecule gives the impression of a ring with a cavity inside. The bright spots in each ring show that hydrogen atoms are directly bound to the carbon atoms in the benzene ring. Figure 1c represents a “chicken-wire” (honeycomb) configuration. The chicken wire structure comprises of a six-fold ring of TMA molecules with the impeccable organization of hydrogen bonds (H-bonds) having a length of 2.92 ± 0.2 A° within the range of OH-O bonds 2.7 A° –3.1 A° with a packing density of 0.007 molecules/A°^2^. The H-bonds between the TMA molecules were specified via red outlines (Figure 1c), having a length of 2.92 ± 0.2 Å. In chicken-wire assembly, every TMA molecule is part of three adjoining rings. In chicken-wire configuration (Figure 1d), the STM studies presented a self-assembled complex assembly comprising six-fold rings. However, in the flower structure, a compact packing of the six-fold rings takes place. The hydrogen bonding takes place within the rings between three molecules (Figure 1d). As a result, the H-bonds possess a length of 2.96 ± 0.2 Å, having a packing density of 0.03 molecules/Å^2^. In both phases, the molecular assemblies were centered on the six-fold ring with a void in the focal point of the individual ring. In the case of chicken-wire configuration, all cavities possess a diameter equivalent to 15 Å. However, in the flower assembly, two types of cavities were present. One exists in the center of the six-fold ring, and the other exists at the points where two adjacent rings touch each other, forming a rectangle void (D = 7.5 Å) on every side of the hexagon [1]. Thus, TMA participates as a host complex with appropriate possible adsorption spots for guest molecules.

Additionally, both TMA phases also act as promising guest molecules. The guest is either located in centric locus concerning the rings bounded with two H-bonds showing stability (the chicken-wire structure), or it may reside flat inside the ring, assemble above (0.05 nm above the ring), or oriented in an upright vertical position (0.4 nm, flower structure). The HG interaction deals with the significant conceptions of supramolecular interaction, which defines the establishment of distinctive assembly developments via non-covalent interactions involving two or more molecules or ions. Previously, with the historical, technological advancement in carbon-based aqueous solutions, there is a growing curiosity in instigating supramolecular HG interacting systems accumulating on dense, smooth planes. The existence of a compact dense plane confirms higher crystallinity in the host complex, facilitating a competent guest entrap. Besides this, it also offers surplus strength and stability to the HG complex through molecular surface interactions. 

## 3. Self-Assembled Isoreticular Host Structures

The exploration of different constituents and their corresponding functions become the primary motivation behind supramolecular interaction investigation. The purpose and behavior of supramolecular assemblies depend on the structure and morphology of crystals. The supramolecular nanostructure manipulation is the critical phase in regulating the properties of these self-accumulated assemblies. The adaptation, modification, and adjustment of voids within porous nanostructures are challenging and demanding in the arena of supramolecular interaction. The fundamental approach involves variation in pore aspects, the organic host system functionality via modifying building block dimensions while preserving consistency in the network topology. Isoreticular word was initially used in metal-organic frameworks (MOFs), which signifies the exceptional dependability of supramolecular synthons. The larger pore size in supramolecular assemblies increases the probability of entrapping bigger guest molecules or larger multiple aggregates of guest capture per void. Organic synthesis is an essential factor in enhancing pore size. In the solid-liquid interface, molecular compactness is principally governed by considerations of shape and size, entitled ‘close-packing.’ This adjacent compact packing is enthalpically preferred because of intermolecular interactions. Consequently, it helps in generating an open porous system at the LS interface, which is dynamically favored because of its lesser adsorption enthalpy. 

Terephthalic acid (TA), a tiny molecule, displays open porous networks through hydrogen bonding (anisotropic forces) and van der Waals interactions (isotropic effects). The equilibrium among long-range anisotropic forces and average range isotropic forces, i.e., vdW interactions, lead towards the transformation of porous configurations in impenetrable dense assemblies except for enthalpy lessening in establishing the porous assembly recompensed via solvent or guest molecules co-adsorption. Moreover, the assemblies of self-assembled complexes were governed by the supporting substrate, solvent, temperature, solution concentration, UHV, or air environment. The initiation of isoreticular host structures involves a systematic in-depth perception of interfacial and intermolecular interactions. Previously, isoreticular host complexes having pore thicknesses up to 7.5 nm have been fabricated [33,34]. In the following segment, we feature some classifications of isoreticular host systems. The H-bonded host structural interactions between carboxylic groups remain one of the repeatedly used molecules because of their resiliency and directional H-bonding. The carboxyl assemblies considerably develop synthons since they can have distinctive ‘‘self-complementary’’ hydrogen bonding capability. The hydroxyl assembly performs as an H-bond donor in these synthons, while the oxygen atom behaves as an H-bond acceptor. A cyclic dimer formation takes place when two carboxylic groups interconnect via two equivalent hydrogen bonds. Besides this, in surface accumulated complexes, different binding assemblies were comprised of trimers or catemers. However, the establishment of accomplishing a 2D (porous) system is not only dependent on the occurrence of a carboxyl group. Minimally, three applicable oriented carboxyl assemblies were prerequisites to produce a protracted linkage centered on H-bonding. Different acids, such as isophthalic acid (ISA), Phthalic acid, TMA, or TA, all possess carboxyl assemblies. Though, among them, merely TMA generates an extensive porous system established via H-bonds.

**Figure 2:**
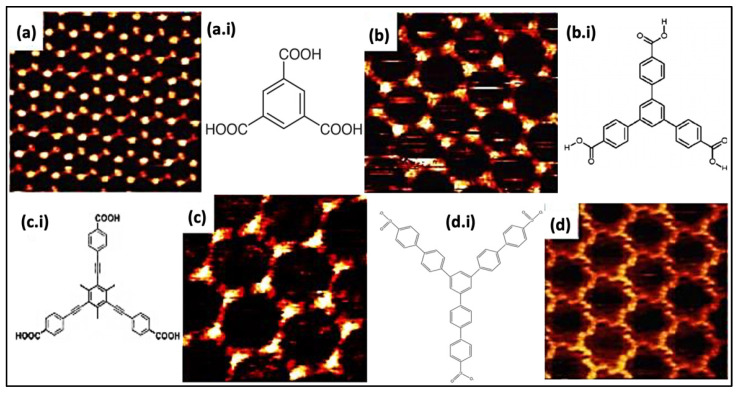
Represents the isoreticular host systems (H-bonding between carboxyl assemblies). STM images of porous coordination establish via (**a**) TMA, (**b**) BTB, (**c**) BTrB, and (**d**) TCBPB. (**a.i**–**d.i**) displays the molecular structure for the analogous porous systems Reproduced from ref. [2] with permission from the American Chemical Society.

The TMA assemblies stabilized via metal-organic coordinate bonds were also narrated previously. The earliest description of the network of nanoporous TMA was conducted under the UHV environment on a solid interface. Many exciting effects were acquired while investigating the self-accumulated assembly at the LS interface. Characteristically, on HOPG substrates, fatty acids were used as diluents. Adding spacers between the central benzene ring and the external carboxylic assemblies enhances host cavities, preserving the elementary three-fold symmetry. 1,3,5-Tris(4-carboxyphenyl) benzene, as shown in Figure 2a,b is a more prominent analogue of TMA, which comprises an additional phenyl insertion between each carboxyl assembly and benzene. Similarly, BTB accumulates itself into a porous honeycomb set-up persistent via resonance stabilized H-bonds analogous to TMA.

The spongy BTB complex has been acquired in both UV and liquid-solid interfaces [35]. It provides superior hexagonal voids (D = 2.8 nm), twice more than a two-fold increase compared to the TMA network voids. BTB exhibits self-connecting physiognomies in three different structural polymorphs, depending on the solvent type, solution concentration, voltage polarity, and temperature [36]. Alternatively, the TMA resembling molecule was also attainable by introducing a phenylethyne spacer between the carboxyl clusters and phenyl rings (BTrB, Figure 2c). The resultant molecular assembly generates a honeycomb void porous system having a diameter of 3.5 nm at a liquid-solid interface Figure 2c [37]. The calculation of Gibbs’s free energy implies that chicken wire morphology (diameter [D]~3.5) is thermodynamically stable and durable in comparison with the ideal honeycomb system (Figure 2d). The larger size of molecular assemblies proves that the molecule-surface interaction is more dominating in the assemblage progression. Besides this, isoreticular linkages centered on TMA comprises alkoxy chains in between carboxylic groups and phenyl ring. Usually, an alkoxy chain is generated via linking up to 10 carbon atoms to form variable void dimensions displaying porous complex. The carboxymethoxy spacer is used to create a TMA network resembling a topological system. However, different derivatives lead towards various void diameters, morphology, or distorted porous linkage depending upon surface adsorption. The orientation deformation and pores distortion depend on two influencing factors: (i) The existence of directional H-bonding between terminal carboxyl clusters, and (ii) vdW interactions between alkoxy chains (exhibit close-packing affinity). These approaches create flexible host linkages centered on carboxyl hydrogen bonding while it does not necessarily resemble TMA isotopologically. The HG interaction of these ‘telechelic’ TMA self-assembling derivatives has been summarized in recent times [38]. The weaker hydrogen interaction also plays an essential part in generating porous networks in molecular assemblies at the nanolevel besides strong H-bonds interaction with the carboxylic assemblies. One such example is the self-compiled anthraquinone molecules on Cu (111) investigated under UHV settings [39,40]. The engender honeycomb linkage is maintained via hydrogen interactions among the carbonyl oxygen and aromatic H-atoms (D~5.0 nm). The important component comprises a trimer of anthraquinone molecules. A stable equilibrium exists due to intermolecular interaction and surface facilitated long order repulsion in the host system, which helps to accumulate this molecular assembly.

In surface-confined linkages in HG relations, guest immobilization is often observed, as the porous host networks directly interact with the guest molecules. One such example is the immobilization of cobalt (Co) molecules in honeycomb host voids, which is due to the confined surface states in which the guest species is entrapped in the center away from the host walls. A steady upsurge in Co molecules on the host surface demonstrated a distinct categorization in which Co guests reside in particular positions within the host cavity. The comprehensive analysis shows that the filling of the locations is directly linked with a filling of electrons into an atomic orbital [41]. The vdW interactions inherently lack the strength and directional capability; however, appropriate molecular schemes were exceptionally operative to generate apparent self-assembly. Close-packed alkyl chains display the ordinarily conferred kind of vdW associations. However, the vdW interaction energy remains characteristically fewer than that of H-bonds. Thus, cooperatively, the interface interactions can compete, including H-bonds.

Moreover, alkanes or alkyl molecular chains interact intensely with HOPG substrate via attractive vdW forces. On graphite substrate, close-packed 2D lamellae formation occurs via linear alkanes, which shows stability due to vdW interactions between molecule-substrate and molecule-molecule associations. Alkanes were strongly adsorbed on HOPG due to structural correspondences between the graphite lattice and alkane molecules. The graphite basal (0001) plane shows a three-fold regularity. Furthermore, in an alkyl chain, each alternative methylene group has a distance of (2.58 Å), nearly equivalent to the in-plane lattice constant of HOPG (2.46 Å). These identical morphological circumstances favour the methylene groups of trans-alkyl chains to settle themselves above a lattice of graphite hexagon cavities. HOPG substrate offers epitaxial equilibrium to alkanes or alkylated molecules. The porous complexes of triangular phenylene-ethynylene macrocycles demonstrate the presence of effective directional intermolecular interactions via van der Waals forces, referred to as “DBAs” [3]. The building blocks of DBAs comprise a firm triangular or rhombic dehydrobenzoannulene core substituted with alkoxy or alkyl chains [19]. The vdW forces play a dominating role in stabilizing the self-assembled network of peripheral chains via surface interactions and developing a strong directional intermolecular bond by distinctive binding configuration commonly known as interdigitation. A fundamental component in the porous honeycomb system comprises a dimer of DBA molecules. The two particles interrelate with each other via vdW forces between their interdigitated alkyl chains. The alkyl chain concentration directs DBA cores distance and hexagonal void size within the self-assembled setup [42]. Direct methods to develop large porous networks were easily achieved by enhancing chain dimensions. Besides this, concentration reliance also influences the development of the pore in the DBA self-assembly network. The solution concentration influencing the structural establishment of molecular assembly at the LS interface was first discovered in DBAs [43]. The high concentration develops dense and less porous molecular assemblies, low levels establish into porous honeycomb networks, and average concentration leads to both phases’ coexistence.

**Figure 3:**
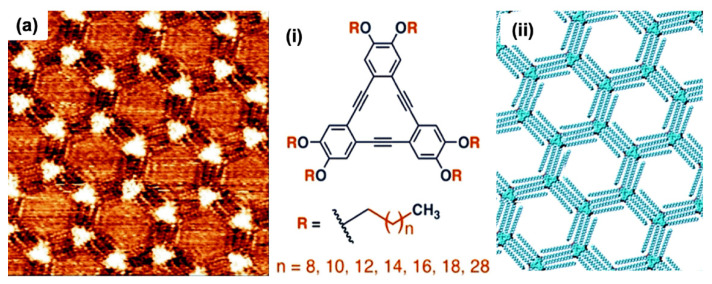
The isoreticular host networks (vdW interactions between alkyl chains). (**a**) The STM images of porous honeycomb linkages generate via DBA. The inset displays the (**i**) molecular configuration (**ii**) models for the corresponding permeable systems, respectively Reproduced from ref. [3] with permission from the Royal Society of Chemistry.

Besides this, the molecular adsorption of DBAs also differs influenced by solution concentration. At a low solution concentration, the alkyl chain adsorption takes place on the surface of the porous honeycomb structure. One or more alkyl chain desorption occurs at the surface in high concentration, establishing a dense packing. However, the concentration reliance evolves due to molecular densities and corresponding stability between two phases. As a result, the network development relies on adsorption energy per unit area at high concentrations, leading to a close-packed dense setup. On the other hand, some molecules at low concentrations accessible to overlay the surface decline, lead to the configuration of porous honeycomb linkage to enhance adsorption energy/molecule. The transformation from porous to dense morphology is also governed by alkyl chain length. In honeycomb structure, a linear relationship exists between surface coverage and the concentration for DBA having smaller alkoxy chains [3]. In the case of longer alkoxy chains, it follows exponential relation. DBAs with smaller chain lengths favorably establishes in porous set-ups. Although, energy difference upsurges by growing chain length, supporting a close-packed non-porous DBAs assembly possessing extended chains [33]. The molecular assembly reliance on solution concentration laid the basis of isoreticular HG linkages centered on vdW interactions between interdigitating alkyl chains. In the alkyl chain, the length augmentation of 1.25 Å per methylene group generates a linear increase in pores hexagonally. A porous network on graphite was fabricated using this strategy with a pore size varying from 2.6 to 7.5 nm [33,44] (Figure 3a).

Thus, DBAs symbolize a standard illustration where synthesis, molecular patterning, and supramolecular surface science approaches have been efficiently used to comprehend functional surface linkages. Therefore, the host complexes immobilize diverse guest species in (hetero) molecular clusters or large shape-persistent macrocycles [33,44,45]. The fundamental characteristics of host linkage were modified to the regularity and dimensions of guest molecules, which can easily be confined. The size variation of cavities is centered on the appropriate choice of the alkyl chain. This is an influential tactic to isolate guests because of fragile networks. However, the efforts to steady them are still in progress. Such as the establishment of considerable pores restrained molecular complexes of a triangular-shaped dehydrobenzo [12] annulene derivative (D~7.0 nm) permitting adsorption-desorption and rotational dynamics of an individual guest molecule [33]. The study of 2D self-assembly comprising non-planar polycyclic aromatic cores and varying alkyl chain dimensions was also performed. The molecular alignment and alkyl chain control the assembly configuration, displaying numerous well-organized adlayers possessing lamellar, hexagonal honeycomb, or pseudo honeycomb assemblies [46]. It shows that molecular dynamics play a potential role as a fundamental entity for nanodevices via self-assembled structural design on 2D compact planes. As described, DBAs generate porous 2D molecular assemblies with hexagonal lattices via vdW interactions between interdigitated directional alkyl chains at LS interfaces. Previously, DBA molecules were comprehensively explored as a rigid core building block due to its adaptivity and adaptability, such as controlling pore dimensions via varying alkoxy chain size, using parity influence by selecting odd or even alkoxy chain numbers, establishing chirality in supramolecules via inducing stereocenters into alkoxy chains or introducing functional groups at alkoxy chain ends to chemically modify the pore interior for guest molecule selective adsorption. The STM enabled researchers to attain information regarding molecular interactions at a nearly atomic-level precision [47].

## 4. Metal–Ligand Coordination; Metal–Organic Framework (MOF)

The metal-organic coordination assemblies are constituents consist of reticular metal centers and organic linkers [48]. These binary components bind together through metal–ligand coordination interaction generating porous organization or metal–organic frameworks (MOF), exhibiting enormously adaptable topologies and potential applications [49]. Thus, 3D interaction has been prolonged to 2D to fabricate MOF on a substrate whose topology, configuration, property, or alignment can be tailored via external stimuli for gas sensing or in optoelectronics [50]. Different MOFs comprising 3D, 2D, bulk, or ultra-thin nanostructures can be synthesized, such as metal-organic, surface-confined, or metal-organic graphene coordination networks. MOFs specify a substitute route to stimulate porous 2D complexes. These interactions were corresponding to H-bonds due to their interface directionality. The metal-ligand coordinate bonds are comparatively inconsistent and unsteady in comparison to covalent and hydrogen bonding. Previously different Metallo-supramolecular complexes have been investigated under UHV conditions. A unique approach involves the merging of preferred organic ligands with metal centers. High purity metal oxides were thermally evaporated to acquire metal centers or extracted from metals as metal ad-atoms. Generally, the metals comprise Au, Cu, or Ag having rock crystal facades, whereas carbon-based ligands were established on carboxylate, pyridine, pyrrole, hydroxyl, or carbonitrile functional assemblies [51].

A porous honeycomb network establishes via dicarbo nitrile polyphenylenes (NC-Phn-CN, where n represents phenyl clusters), creating an exceptional illustration of isoreticular host set-ups centered on MOFs coordination. Dicarbonitrile-polyphenylenes varying in dimensions (1.66 nm (*n* = 3) to 2.96 nm (*n* = 6)) have been investigated on Ag (111) substrate [52]. The investigational procedure implicates transferal of the submonolayer extent of the organic ligand on Ag substrate monitored through its exposure to cobalt atoms beam at 300 K. The bringing together of carbonitrile clusters and cobalt atoms initiates the formation of a honeycomb structure. The NC-Phn-CN ligands align at hexagonal voids walls; however, cobalt atoms occupy the position of vertices. Individually nodal Co atom synchronizes with three ligands. These MOFs correspond to the bottom lying Ag framework in which the alignment of linkage is controlled through polyphenylene interaction, which acts as a backbone. Utilizing this approach, isoreticular honeycomb develops permeable linkages with void dimensions alternating from 4.2 to 6.7 nm on Ag supporting substrate [52]. The accumulation of planar, triangular, square, or hexagonal 2D monomers directs towards prolonged supramolecular organic frameworks (SOFs). The framework link ensues between identical building blocks; it may comprise different dowel alike ditopic monomer. Generally, the interaction between homomeric or heteromeric entities was attained via encapsulating the interactive sites through the macrocyclic host. These types of SOFs exhibit various HG interacting frameworks. Different aspects of HG setups have been explored previously. This kind of porous 2D network can be easily prepared using water as a solvent, forming an extended range of well-ordered, upright layers spreading various square micrometers [53].

Hong-Cai Zhou [54] assimilates Valence Tautomerism (VT) coordination in MOFs. The structural characteristics of VTMOF (dinuclear cobalt-catechol groups and solvent reachable cavities) are innovative, establishing a new direction in this arena. VTMOF displays the response to external temperature and various solvents such as n-butanol, tert-butanol, or isopropyl alcohol. These kinds of supramolecular assemblies are beneficial in tunable sensing behaviors [55]. Recent studies of 2D MOF, such as a study of surface-mediated reactions of 1,3,5-tris(4-mercaptophenyl) benzene on copper and silver, showed closely dense trigonal assemblies. However, thermal treatment exposes distinctive transformations on a copper substrate. A medium annealing temperature (~150 °C) shows the occurrence of dual dissimilar porous linkages. On silver, increasing temperatures (~300 °C) were a requisite to persuade structural variations of 2D honeycomb MOFs on the epitaxial graphene substrate [56,57]. Additionally, the mixed-valence MOFs molecules demonstrate the probability of developing the charge transference mechanism to generate molecular constituents used in microelectronic strategies such as trimetallic molecule Fe^3+^, associated with the central benzene at the 1, 3, and 5 sites displaying a stable asymmetric electronic structure. The electronic structure is subject to tip-dependent variation in intramolecular structure for the Fe^3+^ mixed-valence system [58]. 

Thus, MOF was investigated a lot by different research groups due to its practical applicability in device fabrication such as photovoltaics, semiconductors, hydrogen storage, water purification, drug delivery, or sensor devices for detecting and removing environmental pollutants [59,60] such as the study of 2D MOF of thiolate copper coordination bonds under UHV conditions on Ag (111) and Cu (111) substrate. A room temperature study reveals similar trigonal topography and lattice parameters. However, annealing generates two varying porous nanostructures on Cu (111) at ~150 °C. On Ag, (111) structural transformation observes up to ~300 °C) [56,57] or the exploration of aromatic carboxylic acids having directional noncovalent bonding on Au and HOPG substrate generating 2D MOF possessing intermolecular or molecule substrate interfaces, influenced by bonding formation or molecular arrangement [59], or the study of a low symmetric aromatic carboxylic acid possessing meta-carboxyl groups structured by pyridine derivatives [61]. Tha halogen-adatom structural transformation in 2D molecular grouping shows non-covalent interactions and catalytic responses on a metal substrate [62]. Governable development of numerous organometallic assemblies via bond activation approach in the stepwise process [63] or creation of 2D MOF via dehydrogenation of aromatic amines on the Cu (111) or co-adsorbing organic ligands and metal atoms [64] for the forthcoming expansion of nanoelectronics and 2D crystal engineering. Thus, molecular orbitals and spin-state of the complex can be engineered via selecting metal atoms (iron or nickel). The high spin Fe composite complex possesses a single occupied delocalized orbital, having huge spin-splitting for engineering complexes as modular building blocks in molecular spintronics [65,66].

Thus, MOFs fascinate due to their crystalline configurations, high permeability, porosity, absorbency, and tunability due to their pore size, dimension, and structure from the microporous to mesoporous scale. Recently, alteration of MOFs has been determined to develop their effectiveness for specific applications and broaden their applicability in numerous research areas, i.e., catalysis [67]. The industrialized and agronomic expansion is developing progressively, generating severe heavy metal ion pollution, causing high toxicity to humans and injurious consequences on the environment and natural balance [68,69]. The 2D/3D MOFs or organic-inorganic complex-composites attract unconceivable responsiveness to overcome these challenges due to MOF architecture, nanocrystalline particles, core-shell structures, or hierarchical porosity in terms of composite materialization with numerous constituents having a unique design and synthesis for removal of heavy metal atoms from the water via low-cost efficiency, scalability, multiple metals removals, or reusability.

## 5. HG Interaction: 2D Supramolecular Networks

**Figure 4:**
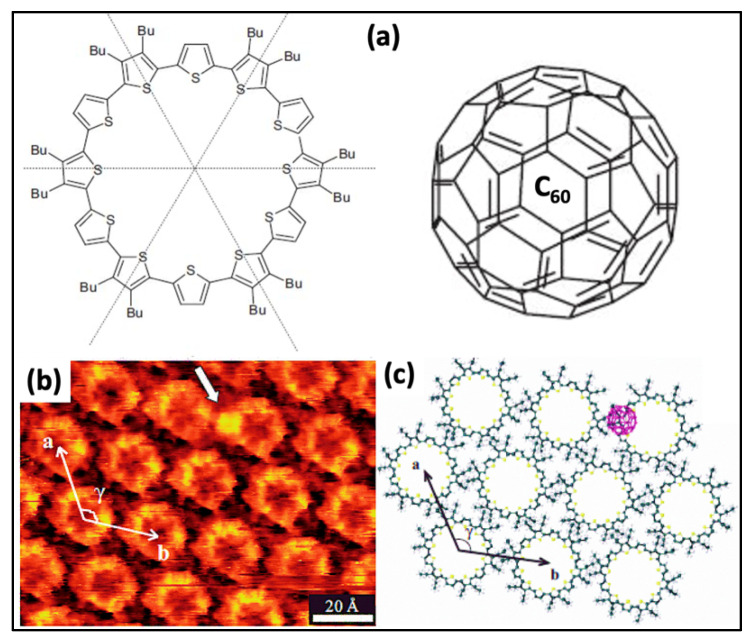
(**a**) Conjugated macrocycle C [12]T (1) and C60. (**b**) STM image representing C12 monolayer, comprising C [12]T–C60 composite (Vbias = −700 mV, It = 44 pA) (**c**) schematic model representing the hexagonal arrangement of a closely packed monolayer of C [12]T–C60 complex Reproduced from ref. [4] with permission from the Wiley-VCH Verlag GmbH & Co. KGaA.

HG interaction on a solid-liquid interface has been observed using hosts having intrinsic or extrinsic porosity. The host cavity possessing intrinsic pores depends on the synthesis, while it is a repercussion of supramolecular self-assembly in the case of extrinsic porosity. However, both of these categories comprise extended 2D networks. The restriction of host networks retaining intrinsic voids is the main building block for the organic synthesis of HG molecular assemblies [70]. Additionally, in host molecules, introducing a preferred alteration in intrinsically porous assembly is complicated. In the following section, we try to elaborate HG interaction via porous organic molecules. The individual building units with extrinsic cavities are not capable of entrapping guest molecules. The intrinsically porous host’s networks often intermingle with guest species in solution or the surface interface. A high correlation exists linking these two components together, which develop precise guest adjustments with one stoichiometry ratio. Characteristically, tiny molecular entities, i.e., cations or C_60_, contribute as a guest; on the other hand, bigger molecules hexa-peri-hexabenzocoronene (HBC) or macrocyclic peptide valinomycin can also restrain by large macrocyclic voids interface. Cyclothiophene macrocycle generates a host system in which C_60_ entraps establishing HG linkage at the organic LS interface, as shown in Figure 4a. Besides π-π interactions, the HG composite system becomes stable by donating and accepting electrons. C_60_ serves as the electron acceptor, and the cyclothiophene macrocycle is electron-rich, so it acts as a donor. The donor and acceptor interactions are significantly peculiar. Generally, C_60_ molecules preferentially occupy the rim position of the macrocycle rather than accumulating the covalent void (Figure 4b,c). The electrostatic force exists between C_60_ and cyclothiophene macrocycle, so the stoichiometry at the stratum surface is always 1:1 on HOPG.

The C_60_ guest binding to one side of the circumference considerably modifies the macrocycle electron density generating an intrinsic dipole. Thus, the opposite rim end develops electron deficiency that cannot bind additional C_60_ [70,71]. Carbon-based macrocyclic have also been investigated, which behaves as hosts in LS interactions in the HG system. Besides their guest binding proficiency, anomalous results were also attained due to surface confinement and close packing. A crown ether substituted phthalocyanine derivative formulates a well-organized arrangement on a gold substrate capable of interacting with Ca^2+^ ions. Though, regardless of the accessibility of tetra linking locations per molecule, only two sites of crown ether show the presence of Ca^2+^ ions due to electrostatic repulsion. After occupying the remaining left crown ether moieties, bound Ca^2+^ ions interact closely to crown ethers of adjoining hosts that entrap ionic molecules as guests.

Moreover, crystallographic coordination also impacts the ion binding studied on a gold substrate. Recently, sodium, potassium, hydrogen, and cesium ions interacting with dibenzo crown ethers have also been investigated through STM on numerous substrates. Furthermore, anion binding is also explored via STM [72]. Recent illustrations take account of iodide binding to a tricarbazolo and triazolophane macrocycle bindings of hexafluorophosphate anions to ‘cyanostars’ (a kind of macrocycle) [72]; both explored on the HOPG substrate. In the illustrations mentioned above, the anion binding stimulated advanced order macrocycles stacks, signifying the dynamic part of a guest molecule. The structural design of a host molecule established an enormous configuration persistent macrocycle. Highly advanced imitation methodologies have delivered access to huge metallic carbon-based macrocycles [70,72,73]. However, the LS interface is preferably appropriate for generating HG assembly of these sorts of composites.

**Figure 5:**
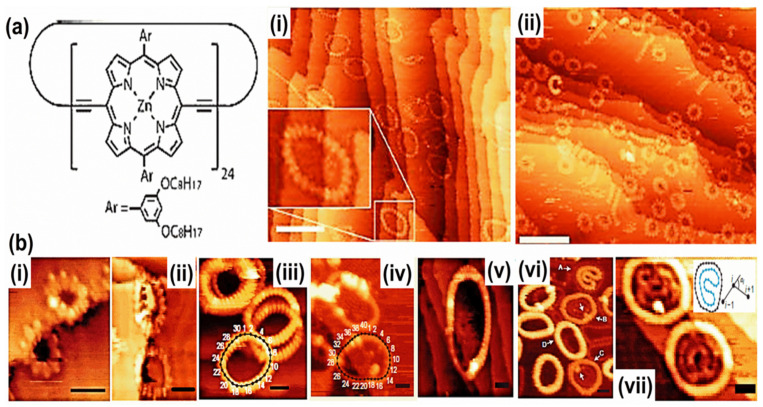
(**a**) Schematic model of c-P24. (**i**) c-P24 on gold deposited from methanol/toluene 1:3, 5% pyridine. (**ii**) c-P12 on Au (111) deposited from methanol/toluene 1:3. (I_t_ = 30 pA, V_bias_ = −1.8 V, −2.0 V, scale bars: 20 nm). (**b**) STM images of nanorings deposited on gold surfaces under UHV. (**i**) c-P10, (**ii**) c-P20, (**iii**). c-P30, (**iv**). c-P40, (**v**). c-P50, (**vi**). Nested nanoring complexes of c-P30 (the labels A, B, C, D in (**b**(**vi**)) represent nanorings structure; A is a ‘double-in-single’ nested structure consisting of two stacked folded rings inside a single open ring. B and C have nested ‘single-in-double’ structures consisting of a single folded ring inside a two-layer stack of open rings. Structure D is a triple-stack nanoring). (**vii**). Nested nanoring complexes of c-P40 consisting of single-height inner rings within double-height outer rings. Inset represents a schematic of a nested c-P40 structure. Reproduced from refs. [5,6] with permission from the American Chemical Society and Nature Publishing Group respectively.

Currently, progression in the synthesis of high molecular weight complexes, i.e., electrospray ionization, has unlocked a novel leading-edge for HG interaction under clean UHV environments. The LS interaction is preferably appropriate for the surface assemblage of these compounds. A distinguished approach regarding macrocycles was established using vernier templating methodology [73]. The approach has been used to fabricate enormous cyclic porphyrin polymers with varying diameters from 4.7 to 21 nm (Figure 5a) [6]. In the UHV environment, electrospray ionization permits the synthesis of huge molecules and their successive configuration on gold, which is accomplished via STM. The surface-adsorbed assemblies of a nanoring having porphyrin units in the polymer expose columnar stacks up to four stratums. These nanorings confine C_60_ in the vacant covalent voids. The layer numbers in the stack influence the confinement of C_60_ [5,70]. A bigger nanoring with ≥30 repeat units demonstrates a distinctive supramolecular ‘nesting’ in which one molecule adsorbs as a folded ring inside another circular nanoring. This kind of auto HG performance was witnessed under UHV conditions along with the solution-solid interface, as shown in Figure 5b [5,70]. Previously, 2D H-bonded supramolecular complexes were extensively investigated through structures having hydroxyl, carboxyl, and amino groups [74]. STM experiments were also conducted to demonstrate supramolecular configurations formed by triacontanol (CH_3_(CH_2_)_29_OH), ISA derivatives, and 1-octadecylamine (CH_3_(CH_2_)_17_NH2) [75]. The stable lamellae develop via H-bonds, and the interactions exist among the extended alkyl branches and substrates. Similarly, 2,6,10-Tricarboxydecyloxy-3,7,11-triundecyloxy triphenylene shows a symmetric triphenylene derivative having tri carboxyl clusters [76]. Previously, experiments were also performed to establish a hexagonal framework via six molecules, and the seventh molecule occupies the position at the octanoic acid and graphite interface [77]. Thus, 2D porous systems are often predictable to restrain guest species dependent on physiognomies of the host cavities, i.e., dimension, profile, and regularity [78,79].

In a bulk self-assemblage of molecules, the unpredictable entropic aspects that lead towards the formation of discrete, porous structural design are often indefinable. The 2D confinement contrary to the surface, restrains various translational, rotational, or vibrational degrees of freedom, permitting well-organized porous structure development. Numerous HG coordination under ambient conditions were explored at the LS interface due to the ease of feasibility experiments. Additionally, it is easy to monitor and control the dynamic aspects of HG interactions at the LS interface. The exchange dynamics and integration of guest molecules in the interior of distinctive host cores have been described via time reliant on STM imaging. These analyses describe the dynamic features of host–guest interaction. In addition, it discloses vital information about the mechanical characteristics of the binding process [80]. In the UHV environment, sometimes HG interaction is delimited and sublime due to the guest constituent part, which is interrelated to its molecular weight, such as the sublimation of C_60_ as a guest constituent.

## 6. Guest-Templated Host Networks

A distinctive characteristic of the HG system is that guest persuaded evolutions occur at the LS interface in host complexes. The guest’s molecules often show an intricate and complicated character, rather than simply submissively inhabiting the voids and open cavities within a host network. The dynamic guest stimulates structural alterations within the host framework reforming 2D configurations [81]. The process is analogous to the induced-fit mechanism perceived in bio-enzymes. The enzyme activates sites that are initiated when it interacts with the substrate. The earliest illustration depicts the self-assembled non-porous arrangement transformation to a porous one in consequence after the addition of a guest molecule. The HG complex in which alkoxy substituted DBAs having *n* ≥ 14 or 16 exhibits compactly packed non-porous linkage comparatively at extreme concentrations. The surplus coronene (COR) addition in a solution generates HG assembly, which directs non-porous assembly transformation to porous honeycomb configuration in which COR molecules employ the guest voids. It is found that planar guest species (strong π conjugate) persuade the dense-to-void conversion regardless of symmetry. The dense host complex cannot be impacted by the non-planar or smaller guest species. Thus, it specifies that porous assembly shows thermal stability via an increase in free energy. Adsorption of guest species takes place by overcoming the inherent energy linked with large cavities [82]. Besides this, the guest stimulated variations are not constrained to vdW interactions on host structures. The self-assembled flower assemblies were generated via C 3-symmetric aromatic carboxylic acids with meta-carboxyl groups having two cavities with different dimensions. The HG coordination in co-assembly displays no molecular discrimination for the selection of a guest entity.

A close-packed parallel configuration formation occurs due to adsorption at the LS interface (i.e., quaterphenyl tetracarboxylic acid (QPTC)). The energetic structure preference can be altered by adding a guest entity, i.e., coronene generating a 2D Kagome complex. In recent times, in a 2D H-bonded porous complex, entrapping C_60_, it was observed that the growth initiation of second layer in the host setup develops in the orthogonal direction to HOPG substrate. The QPTC or TPTC generates a cavities complex possessing hexagonal voids via in-plane H-bonding between carboxyl groups. Adding C_60_ saturated solution to the TPTC complex encouraged bilayer TPTC development. Bilayer templating shows advancement after the C_60_ addition. The upper stratum is persistent via HG interactions with C_60_ and π stacking with the lower TPTC stratum having interactions with HOPG substrate. The self-energetic environment of robust LS interface, TPTC-C_60_ dual-layer complex, transforms to TPTC–COR single layer complex via adding COR molecules. COR is a favored guest species due to its increased adsorption energy, which helps them accumulate in the voids. This organization exemplifies a significant phase concerning 3D structural design founded on 2D configurations [81,83]. Similar alterations have been described in structures that become stable via equilibrium between diverse supramolecular interactions. The guest-induced energetic HG interaction comprises a supramolecular complex of alkoxy substituted ISA. The generated complex possesses equilibrium due to vdW interactions among interdigitating alkoxy chains (developing a compact network) and H-bonding between carboxyl groups (establishing porous structure). 

## 7. Multicomponent HG-Systems

Previously, numerous HG structures comprising either porous host network or guest class possess more than two constituents having different building units. These intricate hierarchical assemblies at the LS interface require comprehensive detailed study. Stable host complexes having permanent voids, i.e., host cavities generated by hydrogen bonding, show the potential capability to entrap guest entities. However, tunable host networks persist via vdW collaborations, which offer extraordinary selectivity in comparison with enzymes. The DBA derivatives exploration has been at the forefront of multicomponent HG systems. The HG system has multi constituents comprising of polycyclic aromatic hydrocarbon nanographene (NG), which serves as guest species for DBA derivatives (varying chain dimension (*n* = 8, 10, 12, 14, 16, 18)). A single NG guest molecule may entrap one or more than two molecules. Nearly six triangular NG guest species can easily immobilize in the host void, relying on the void dimensions accessible by the DBA host network. The distorted hexagon’s existence in the interior of the host complex displays the host network flexibility, which experiences minor distortion to accommodate the guest’s species. The intricacy of hierarchical self-assembly was advanced in a three constituent HG structural design accumulating at the LS interface. The interlinking of the guest species is centered on dimensions and shape. A heteromolecular guest species comprising ISA or COR, can restrain in the cavity of DBA-OC10 derivative which provides the host system. It is a unique arrangement in which guest influence occurs at two different stages. Firstly, ISA itself did not generate cyclic hexamers at the LS interface. Still, it accumulates in a compactly dense crisscross assembly dominated via hydrogen interactions between carboxyl clusters. Second, a COR molecule entraps in the ISA cavity via a hydrogen-bonded hexamer. It also shows concentration dependency, i.e., DBA-OC10 formulate in compactly dense assembly at LS interface (1-octanoic acid/HOPG). COR–ISA heteroclusters generate cavities. Adding a complex solution of COR–ISA to a preassembled complex of DBA-OC10 gives rise to the morphological transformation from dense to porous network, having identical configuration and regularity.

Analogous experiments were performed by premixing three constituents together via the drop-casting method on HOPG substrate. An intricate four-component HG structural design having 2D supramolecular DBAs was also generated. Experiments were also performed to establish a geometrically unique host system harvesting dual guest molecules. Rhombus designed bis-DBA derivatives generate Kagome’s complex, producing spatially organized hexagonal or triangular cavities at 1-octanoic acid/HOPG interface. Though, bis-DBAC12 cannot reform the Kagome complex because of comparable dimensions of hexagonal cavities. Tri composite system consists of COR and ISA solution generates hexagonal voids once dropped slowly. The addition of triangular guest species, i.e., triphenylene, generates multicomponent 2D HG assembly having trilateral cavities. An appropriate concentration control leads to developing this four-component configuration spontaneously at the solvent/graphite interface. The precise size equivalency of guest molecules to dual pores is essential to attain stable 2D self-assembly. Monitoring numerous compounds assembly on metallic substrates is quite problematic in comparison to HOPG due to enhanced diffusion. Three-component HG coordination establishes a unique supramolecular assembly on Au (111) plane. DBA host networks possess chirality. In the DBA host complex, the circumference of individual hexagonal void adsorption on the surface leads towards chirality, with two distinctive interdigitation motifs labeled as (−) and (+). The interdigitation motifs amalgamation covering a discrete cavity generates either a chiral or achiral void of the host network. The host network possessing chiral cavities did group together in 6 indistinguishable interrelated motifs. Achiral cavities comprise 3(−) and 3(+) interdigitation motifs arranged in alternating orientation. The intricate complex of DBA–COR–ISA shows an orderly framework of chiral and achiral voids on a gold substrate. The analogous system generates on graphene substrate, having merely chiral voids. An inimitable assembly was observed on gold substrate interrelated to a reduced energetic preference for chiral pores compared to HOPG and improved diffusion barriers for guest species. In the case of guest molecules, improved diffusion barriers permit the guest entity to initiate nucleation to formulate achiral cavities, leading towards superlattice configuration.

As demonstrated in the examples mentioned above, supramolecular interactions, dimensions, and structure play an important, significant role in most design approaches. Besides this, the particular stereochemical organization of binding spots commands a selection of organic assemblies. Optimum intermolecular or interfacial interactions help guest species to occupy host setups, displaying that host–guest arrangements can be appropriately regenerated. In surface restrained HG networks, the selection criteria may be deliberated via various situations. Firstly, the host setup presents merely a single binding position, and it interacts with a single guest molecule when a lot of guest molecules present in the framework. In the next process, the host link proposes binary categories of binding spots, though (i) entrapping a single guest particle in the merely single pore or (ii) traps dissimilar binary guests in two kinds of voids, showing a self-sorting phenomenon. This guest selection associating via host linkages is significant in upcoming applications, i.e., detecting molecules. In an open host network, chiral DBA (c-DBA) derivatives adsorption formed through structurally corresponding achiral DBA (a-DBA) demonstrates adsorption of the first type of interaction [84]. When (c-DBA) and (a-DBA) accumulate at the LS interface, c-DBA derivative co-adsorb and modify the chiral equilibrium of network. It is known as the “sergeant(S)–soldiers(s)” attitude, where the chiral DBA influences the supramolecular network. Consequently, a combination of c-DBA-OC12(S) termed as a sergeant and DBA-OC12(s) termed as a soldier, direct towards the growth of porous system principally comprising clockwise (CW) nanowells [7]. The chiral DBA molecules, besides adsorption as a part of the network, also occupy the voids of the porous system. The c-DBAs demonstrate a noticeable trend to adsorb in nanowells, i.e., c-DBA-OC12(S) persuades the establishment of CW nanowells. It favorably adsorbs as a guest by adapting a windmill-like confirmation by the in-plane curving of its chiral chains. The c-DBAs adsorb as a guest in comparison to a-DBAs, since c-DBA configuration permits enhance vdW interaction of the substrate via bending chiral methyl clusters away from the substrate. Molecular dynamics display favorable vdW interaction between alkyl chains of the chiral guest and host system. Moreover, nanowells (CW) of a host system did not show similarity with the windmill resembling the assembly as possessed by a guest (CCW) [7,84].

A case in point of the second type was demonstrated by HG assemblage between Bis [3,5-diacidic] diazo-benzene (NN4A) derivative and fullerenes (C_60_ or C_80_). NN4A displays porous Kagome linkage via H-bonding exist in ISA entities. Side selectivity is not observed in C_60_ adsorption; C_60_ is entrapped by either hexagonal (type A) or triangular (type B) voids. Bigger fullerenes, i.e., C_80_ or Sc_3_N@C_80,_ entirely inhabited greater voids comprising hexagonal shapes exhibiting site-selective linking. Additionally, Sc_3_N@C_80_ possesses enhanced electronegativity due to the captive conductive atom, which enhances affinity with the host complex, presenting a durable, well-organized HG system [85]. Xiaoyang Zhu and his group mates investigated the NN4A molecule via STM and AFM due to its photosensitive azo cluster and carboxylic assembly owning H-bonding influence. At the same time, porphyrin groups comprise diverse statistics and spots of carboxylic acid clusters considered as another constituent. They experimentally perceived that porphyrin implements diverse adsorption patterns due to carboxylic group impact, leading to structural transformation to generate various succeeding co-assemblies [86]. 

In recent times, the self-arrangement of guest molecules via the DBA host system is maximally explored. Host linkage comprises periodically functionalized cavities of varying sizes via tailoring DBA derivatives. The approach is based on DBA derivative having a single functionalized alkoxy chain while the other five alkoxy chains remain as they are, without having a functional group, which possibly leads towards the establishment of two kinds of configurations. In configuration one, the functional assemblies are at a random position in the nanowells. However, when functional groups occupy the same nanowell, then the subsequent complex formulates intermittently functional host voids. This strategy develops ISA’s capability to formulate H-bonded cyclical hexamer. Iso-DBA accumulates itself at the LS interface entirely upon annealing. The system shows hexagonal voids in which every single nanowell was comprising the cyclical hexamer of ISA units bounded by six non-functional nanowells. The nanoscale separation of ISA units encompassing voids is preferred due to the enthalpic gain related to the H-bonds creation between six ISA units. The nanowells formulate at irregular intervals on the substrate, having different dimensions. These nanowells can easily immobilize COR or a huge aromatic molecule depending on the site. Similarly, butadiene-bridged planar molecules also show analogous site-selective guests in a single-layer network [87]. Thus, self-recognition and dynamic self-organization are also very important, considering factors for assemblage supramolecular coordination. Earlier, at the molecular level, it is slightly difficult to access the information. However, recently different researchers investigate the mixture of organic ligands using copper as supporting substrates such as linear bis-carboxylic acids and bi-pyridines [88], [oligo(2,2′)-bipyridine] composite from helicates self-assembly [89]. These dynamic supramolecular self-assemblies help to design automated structures comprised of amalgamations and are proficient in instinctively developing distinct frameworks via self-processes.

## 8. Stimuli-Responsive HG Systems

The entrapping of guest species and its release in a well order method in voids sustained via host complexes show significant importance for various appliances. Alterations could accomplish the ordered discharge in the host setup activated via applied stimuli such as radiation, annealing, pH, electronic field, or applied polarity. Modification in host structure is a prerequisite to capture guests in a host complex. Thus, triggers applied to the host system are demanding, though, STM permits the time-dependent study of HG structures. In organic molecules, functional supramolecular assemblies were generated via paraquat derivatives or secondary ammonium salts. Different molecules generating host assemblies comprising crown ethers, cucurbiturils, pillararenes, calixarenes cryptands, or calixarenes were used to synthesize self-assemble nanostructures possessing guest species [90]. However, in organic solvents, cryptands possess the greatest stability with paraquat derivatives because of their reorganization and enhanced binding spots. They efficiently facilitate supramolecules coordination in high yields to formulate stimuli-responsive functional structures. A few examples including pillararene-based pseudorotaxane, show photo-responsive self-assembly behavior [91]. The azobenzene containing cryptand generating HG system possesses detectable fluorescent output signals. The cryptand displays switching (ON−OFF) transformation via 2,7-diazapyrenium (DAP) derivatives [92]. Pillar[6]arene dependent photoresponsive HG system can be controlled via UV or visible light irradiation, a phase swapping between vesicle-like and irregular clusters occurs [93]. Pillar[5]arene dependent supramolecular polymers cause quenching and photon-initiated emission. Supramolecular polymeric configurations display analogous assemblies, i.e., “head-to-tail” assemblies, which efficiently declines excimer development. It results in molecule accumulation causing quenching, improving solid-state emission efficacy showing potential applicability in optoelectronic and bioimaging applications [94]. Thus, covalent polymers associated via non-covalent interactions establish supramolecular polymer networks resulting in self-assembled materials having assemblage stability, recyclability, processability, incentives response, or self-restorative ability [95]. The fabrication of ring-in-ring(s) composites displays adjustable multicolor photoluminescence due to hydrophobic interactions linked with a specified entropy change directed to practical applications such as visual displays or multidimensional biological imaging [96]. Many practical, applicable HG relations were recently explored by different research groups, such as HG relation of perethylated Pillar[6]arene with ferrocene derivatives [97]. Supramolecular HG self-organize assemblage of histidine-capped-dialkoxy-anthracene (HDA) forms light stimuli nanoassemblies [98], such as light assisted alterable self-assembly of nanorod supra-structures comprising zinc ions coordination in the presence of complex 4,4′-dipyridine in β-cyclodextrin [99]. Besides this, photocontrol and adjustable secondary self-assemblage of supramolecular nanosheets show drug delivery behavior in biomedical applications [100]. The water-soluble amino pillar[5]arene functional with Au nanoparticles serves as a fluorescence probe for dopamine determination [100]. The pillar[n]arene macromolecule shows stable HG supramolecular switches in solution and on surfaces due to their binding ability of various guest molecules and multipurpose, adaptable functionalization [101]. The photo-responsive composite surface was assembled via adjusting azobenzene-calix[4]arene on a micro-structured silicon substrate using UV light irradiation as a stimulus [102]. SOF luminescent-based chemo-sensors demonstrate selectivity, sensitivity, and applicability comprises macrocycles, polymers, and nanomaterials [103]. Thus, light stimulated organic molecular motors are at the heart of cellular machinery, involved in transforming chemical or light energy into effectual output mechanical effort [104] or thermosensitive behavior of Benzo-21-crown-7 [B21C7] [105]. Thus, introducing the stimuli-responsive functional groups into a supramolecular network is useful to control the dimensions, configuration, and applications via external stimuli, i.e., temperature, light, pH, or periodic and rotational motions at the nanoscale [106,107].

Besides this, the dicarboxyazobenzene discussed previously has also been implemented to comprehend stimuli-responsive host voids in which light acts as stimuli. Dicarboxyazobenzene units were linked to alternating alkoxy chains. A honeycomb complex formation takes place having functional voids encompassing dicarboxyazobenzene constituents as a guest in host cavities. The azobenzene derivatives also show isomerization induced via light [108,109,110]. The host cavities were modified to act in response to light irradiation’s appropriate wavelength. Besides this, the structural variance among non-planar cis alignment and planar trans alignment is used to transform the pore sizes or memory shape devices via photoisomerization. The experimental STM results revealed that guest entrapping cavities comprises hexagonal voids. Azobenzene guest inclined near the midpoint of nanovoids, signifying the development of cyclic hexamer consisting of dicarboxyazobenzene units. This complex preferably entraps an isolated COR molecule per cavity. The guest linking capability was investigated further in the honeycomb complex adsorbed on HOPG via irradiation. When the LS interface is exposed to 320 nm light, dropping the COR solution to assembly shows that two COR molecules are entrapped in the host cavities. The molecular depictions demonstrate that isomerization from trans-to-cis monitored via azobenzene desorption creates a sufficient hole to enclose the second COR molecule. One can modify cis-to-trans isomerization through pores lessening via irradiation [7]. One of the COR guest species eradicates itself from the host void after lessening in pore dimension. Experiments on photoresponsive host structures established on the inherently porous assembly have also been performed. The azo entities that help in isomerization were assimilated into azobenzenophane type 4-NN macrocycle [7]. It does not generate a prolonged 2D system itself. Instead, it immobilizes into the porous self-accumulated complex developed via 1,3,5-tris (10-carboxy-decyl oxy)-benzene (TCDB) at heptanoic acid/HOPG interface. The TCDB complex entraps a monomer or 4-NN macrocycle dimer reliant on solution stoichiometry. STM statistics show that all azo assemblies attain trans configuration. Single stratum (irradiation; 366 nm light) activates the isomerization within the macrocycle rim, directing diverse photoisomers’ establishment. These photoisomers could be recognized from the macrocycle shape [7].

After UV irradiation the trans (t,t,t,t) configuration, (azobenzene units) generates numerous isomers comprising trans–trans–trans–cis (t,t,t,c) or trans–cis–trans–cis (t,c,t,c) isomers. Light-induced stimuli are used to influence the morphology and dimension of the 4-NN macrocycle. 4-NN macrocycle encapsulates and frees COR guest species after light exposure. The trans (t,t,t,t) isomers possess covalent voids that were too tiny to entrap guest species, i.e., COR molecule. Due to this, the accumulation of COR to the TCDB/4-NN structure does not immobilize it. COR guest molecules assemble themselves on a single layer. Experiments were conducted to synthesize supramolecular assembly in which cavities generated by the host network were captured by immobilizing COR guest species when UV irradiated. The macrocycle having parallelogram morphology changes to ellipsoidal after its exposure to light. Thus, photoinduced stimuli alters trans (t,t,t,t) alignment to trans–cis–trans-cis (t,c,t,c) alignment. Thus, the modification upsurges the active space of host cavities, directing them to immobilize COR molecules. Visible light irradiation instigates the reverse alteration to trans configuration. The COR molecules ejected due to contraction in the covalent voids. In supramolecular assemblies, a minor change in applied current or annealing generates alterable changes in voids of the host. An illustration of stimuli receptive HG coordination was validated for hydrogen-bonded complexes such as LS interaction, BTB with octanoic acid investigated on HOPG. As previously explained, BTB formulae into a hydrogen-bonded spongy system such as a honeycomb structure. These porous structures help in the immobilization of planar aromatic guests such as COR or nanographene. One can easily swap between BTB porous and non-porous structures via applied polarity. Temperature rise triggers the conversion of porous linkage into a compact assembly. These nano-assemblies easily shrink or mature via external stimuli in a well-ordered structure. They are essential in emerging stimuli-responsive composites.

## 9. Covalent Organic Framework (COF): 2D Host–Guest Chemistry

The fabrication of 2D COF has grown attention and has been efficaciously accomplished in UHV or ambient environments. COFs are generally acquired as soluble, cross-linked fine particles or stratums, appropriate for device implementation. The building blocks networking via covalent bonds generate robust sheets of material with distinct configuration, composition, dimension, and porosity [111]. Recently, the polycondensation mechanism comprising Schiff bases [112] or boronic acid derivatives [113] was mostly considered. In optimal environments, it can create prolonged porous complexes that compete with supramolecular structures involving the domain size or structural eminence [114]. COFs usually form porous nanostructures via the bottom-up method through molecular interacting building units with a predesigned geometrical structure interrelated through covalent bonding. They deliver an orientational and directional control over the generating building blocks in 2D or 3D. This controlling mechanism enhances the formation of rigid porous nanostructures possessing symmetry and consistency, which helps in enabling the switching of physical and chemical properties. In this section, an overview of rapid developing work regarding COF is discussed, such as various synthesis procedures according to need, i.e., to enhance crystallinity, thin-film formation, or consistency for practical applications including sensing, energy storage, or in optoelectronic devices. Generally, COF was assembled via organic linkers through condensation reactions that take place in a reversible approach. The covalent bonding generates thermal constancy in the assembly. The existence of a reversible nature of applicable coupling reactions enhances the crystalline structure development than the amorphous polymer because of regeneration and connection rearrangement within the lattice. The earliest COFs were generated through co-condensation of boronic acids with catechols to establish five adherent boronic ester rings as a linkage between the building blocks via self- condensing boronic esters to boroxines.

**Figure 6:**
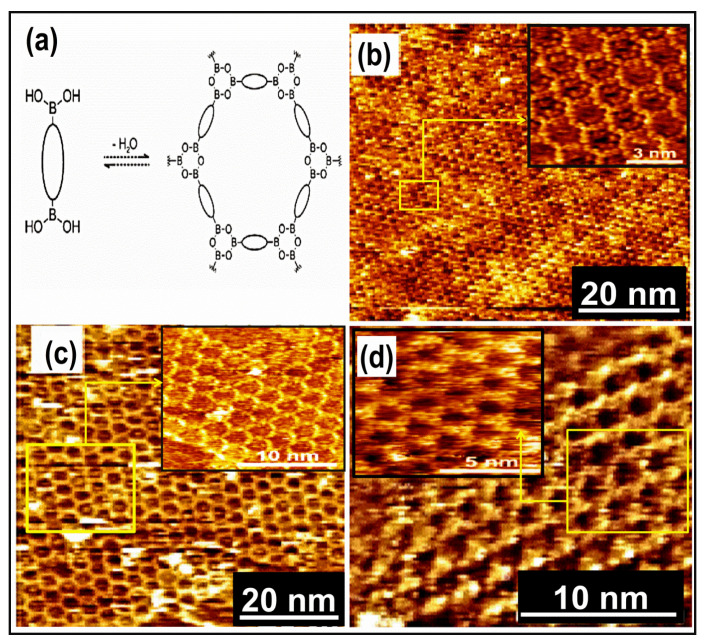
(**a**) Reaction scheme of diboronic acid self-condensation into hexagonal 2D COFs. STM images of 2D COFs resulting from monomers polycondsation, (**b**) 2 monomers, (**c**) 3 monomers, (**d**) 5 monomers (insets demonstrate an enlarged view) Reproduced from ref. [7] with permission from the American Chemical Society.

Covalent organic structures signify a developing class of crystalline porous materials that comprises light elements. In COFs, the building blocks were associated via covalent bonding. Earlier bulk COFs have been comprehensively considered. This kind of network linked via covalent bonding is widely used in prospective applications in gas storage, catalysis, optoelectronics, or photovoltaics [53,115]. However, segregation of monolayers of COFs remains an important subject. The scanning probe approach is used to characterize the distinct layered material. Previously, boronic acids and imines COFs were carried out using STM [115,116]. However, the surface amalgamation of isoreticular monolayer COFs was also validated in recent times [7,117]. The 2D COFs were synthesized by two main methods, including Schiff’s base development and boronic acid self-condensation. These reactions were used to form extended COF with the least defects in reversible layers performed in mild environments. A diboronic acid molecule’s self-condensation directs to developing covalent planes with the hexagonal organization of boroxine (B_3_O_3_) rings, as shown in Figure 6a, interrelated via diboronic acid monomer, which serves as an organic backbone in the assembly. Adding minor water quantity in the reactor commonly helps in reaction reversibility. During the reaction advancement, the water evaporates, which causes an equilibrium shift towards the dehydrated product. A chain of isoreticular 2D COFs was assimilated via the self-condensation of para-boronic acids with growing dimensions of the organic linker from phenyl to quarter phenyl. The covalent host linkages generate voids with dimensions extending from 1.0 to 3.2 nm (Figure 6b–d).

COFs based on isoreticular mixture comprising imine were attained through Schiff’s reaction carried on graphite. It generates covalent layers with well-organized cavities with numerous pores ranging from 1.7 to 3.5 nm [117]. Nowadays, monolayers of imine-based COFs were synthesized at the air-liquid interface. Different difficulties were encountered when COFs were synthesized in bulk or single stratum at the air-liquid interface. It permits the transfer of 2D polymers towards arbitrary planes for comprehensive description at the air-liquid interface, i.e., polymerization of amphiphilic anthraquinone centered monomer via light [118] or COF of imine acquired via covalent interaction [119]. The innovative approach to the manufacture COFs and supramolecular assembly defined above is beneficial in HG chemistry. Additionally, the covalent and non-covalent accumulated host complexes, or inorganics, i.e., nanomesh of hexagonal boron nitride (HBN), exhibit fragile host linkage. Hexagonal boron nitride generates as a nanomesh, similar to a single sheet fabricated on Rh (111). It appears like self-accumulated grooved nanostructured topography having hexagonal-shaped pores (D~2.0 nm). The surface porosity arises due to hexagonal boron nitride interaction with the Rh lattice. The pores’ fringe strongly adheres to metal, while walls of nanocavities are areas where interaction with surface is comparatively feeble. It has been revealed that these nanomesh pores assist as adsorption locations for water, carbon-based compounds, or nanoscale reactors [119,120].

**Figure 7:**
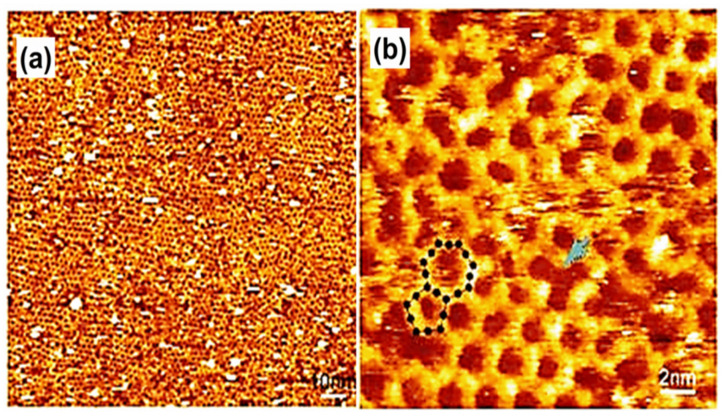
(**a**) Extensive STM image of surface COF1 + 2′. (**b**) STM image of surface COF1 + 2′ a pentagonal neighboring with a heptagonal pore (highlighted by black schematic model), a typical polygon due to incomplete condensation specified by a blue arrow Reproduced from ref. [9] with permission from the American Chemical Society.

On supporting substrate, monolayer COFs physisorbed make a vigorous covalent interaction in HG coordination. Boronic acid acts as a host to immobilize C_60_ through covalent interactions. Condensation of benzene-1,4-diboronic acid is used to generate a single layer of COF. The developed monolayer of COF recommends homogenously dispersed host voids entrapping C_60_ guest molecules having negligible imperfections. In COF, the domain restrictions of C_60_ composite were used to classify grain borderlines of layer substructure [8]. Earlier, numerous co-adsorbed COF systems providing cavities were also explored [8]. Likewise, 2D prolonged aromatic isoreticular Schiff-base surface of COFs was obtained via co-condensation process between aromatic aldehyde and aromatic diamine monomers on HOPG at LS interface with minimal imperfections achieving all-inclusive surface coverage (Figure 7). The tunneling situations were: I_t_ = 0.05 nA, V_bias_ = 0.50 V [114]. The pore dimension of 2D COF assembly is modifiable (D = 1.7~3.5 nm). Besides this, one can reform COF by inducing functional compounds in the precursor (aromatic amine), i.e., inserting methyl assemblies to diamine, as shown in Figure 7 [114]. Likewise, 2D prolonged aromatic isoreticular Schiff-base surface COFs were obtained via a co-condensation process between aromatic aldehyde and aromatic diamine monomers on HOPG at LS interface with minimal imperfections achieving all-inclusive surface coverage (Figure 7b). These defects are very typical defects observable in 2D COF, a single heptagon adjacent to a pentagon. It is analogous to the Stone-Wales defect assembly of HOPG [121]. These imperfections were also noticed in numerous 2D covalently bonded honeycomb linkages [122], though never observed in the noncovalent networks.

**Figure 8:**
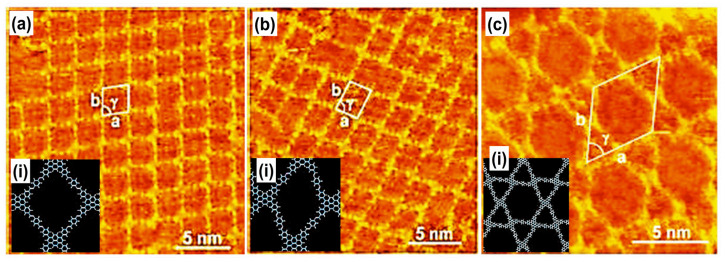
High-resolution STM images and Structural models and measured lattice parameters (**a**) (**i**) rhombus, a = b = 2.5 ± 0.2 nm; γ = 82 ± 2°, (**b**) (**i**) parallelogram, a = 2.3 ± 0.2 nm; b = 2.5 ± 0.2 nm; γ = 71 ± 2°, (**c**) (**i**) Kagome morphological networks, a = 4.7 ± 0.2 nm; b = 4.9 ± 0.2 nm; γ = 61 ± 2°. The imaging conditions were V_bias_ = 700 mV, I_t_ = 500 pA Reproduced from ref. [10] with permission from the American Chemical Society.

Similarly, COFs comprising of a single layer (SCOFs) were also synthesized via tetradentate monomer 1,3,6,8-tetrakis (p-formyl phenyl) pyrene with D_2h_ symmetry and ditopic linear diamine building blocks [10]. Different types of well-organized SCOFs, comprising rhombus, parallelogram, and Kagome networks, were detected. In SCOFs, pore dimensions and sporadic were easily tunable via monomers of a diamine with varying extents. The experimental result shows that two sorts of quadrate linkages were favored at a higher concentration, as shown in Figure 8. However, Kagome networks appear at low concentrations [10] depending on network density. These SCOFs are potential candidates in advanced molecular devices.

These COFs are attaining fascinating interest due to their molecular diversity, biocompatibility, and operation safety. Recently, the boronic ester-based COF was efficaciously synthesized on carbon nanotubes (CNTs) exterior via an organic condensation reaction. It is used as organic anode material for economical potassium ion batteries [123]. It shows ultrahigh potassium storage with high reversible capacities. Moreover, it shows superior performance in comparison with previous organic/inorganic electrodes. P. Peng [124] and his colleagues designed a soluble COF encompassing orderly N-coordinated Fe atoms in the center and conjugated assemblies. This COF possesses a tiny work function (4.84 eV) and better catalytic activity to reduce oxygen. This acquired COF might be employed for zinc-air batteries, producing noticeable efficiency with exceptional strength. In COF, the tussle between constancy, stability, and reversibility depended on reaction settings, leading to chemical durability. As a result, COFs having pervasive structural disorder were acquired [125,126]. Recently, different research groups explored various COF, such as the stacking behavior of 2D imine COFs such as Triazine benzene triphenyl imine (TBI-COF) andTriazine-triazine-triphenyl-imine (TTI-COF) having antiparallel imines as a favored mode) [126]. Pyridine established a photoactive structure where N_2_ exchange in peripheral aryl rings helps in reversing polarization of COF for photocatalytic hydrogen evolution [125]. Similarly, synthesis of photostable 2D azine-linked COFs [127], the formation of nanoporous covalent hexagonal structures through benzenediboronic acid (B-DBA) forming boroxine linked surface COF on Ag (111) substrate [128]. HG interaction of COF with fullerene C_60_ [129] or 2D COFs, possessing an AA stacking arrangement or AB stacking with either serrated or inclined arrangement, are a few examples [111]. The advancement in developing COF functional nanostructures with intriguing properties progresses the strategy of functional nano-architectures due to the amalgamation of the enormous, exposed area, enhancing crystal attributes, variable pore dimension, and perfect molecular structural design [128]. The COFs have shown potential promises for catalysis, water purification, or storing electricity applications from varied applications from operational constituents towards optoelectronic equipment.

## 10. Field-Induced Supramolecular Phase Transitions

In optoelectronics, molecular catalysis, or nanofabrication, tunable supramolecular assemblage has originated numerous applications. The supramolecular assemblies can be intricately planned, smartly organized, and monitored via external stimuli, such as the pH of the solution, applied temperature, electric stimuli, magnetic impact, incident light, or impurity-inducing biomolecules [11]. These phenomena became essential design conceptions in supramolecular interactions or optoelectronics. The swapping of phase via stimuli-induced supramolecules has been comprehensively explored due to its prospective applicability in identifying molecular electronic engineering via surface alterations [130]. T-phase tantalum diselenide (TaSe_2_) inside the 2H-TaSe_2_ crystals’ stratum was assembled via STM at liquid helium temperature. A mechanism involving the phase switching via tip-induced solid-solid interaction was performed to generate T-phase TaSe_2_ nanocrystals ingrained in H-phase TaSe_2_, size varying from 7 to 100 nanometers [131] or the morphology-induced magnetic phase transitions in Fe grown on MgO-Ag (001) [30,132]. Despite its significance, until now, the control of supramolecular self-assembly remains a challenge [133,134]. Previously, many studies have been conducted via external induced stimuli referred to as “STM-induced phase transitions”. These molecules possess the intrinsic intramolecular dipole moment [135,136,137]. Recently, it was explored that phase switching can also occur in TMA, having a chicken-wire structure, an analogue to BTB, as discussed earlier (Figure 1). It is an interesting phenomenon of TMA, as TMA did not own a dipole, nor possesses a charge.

**Figure 9:**
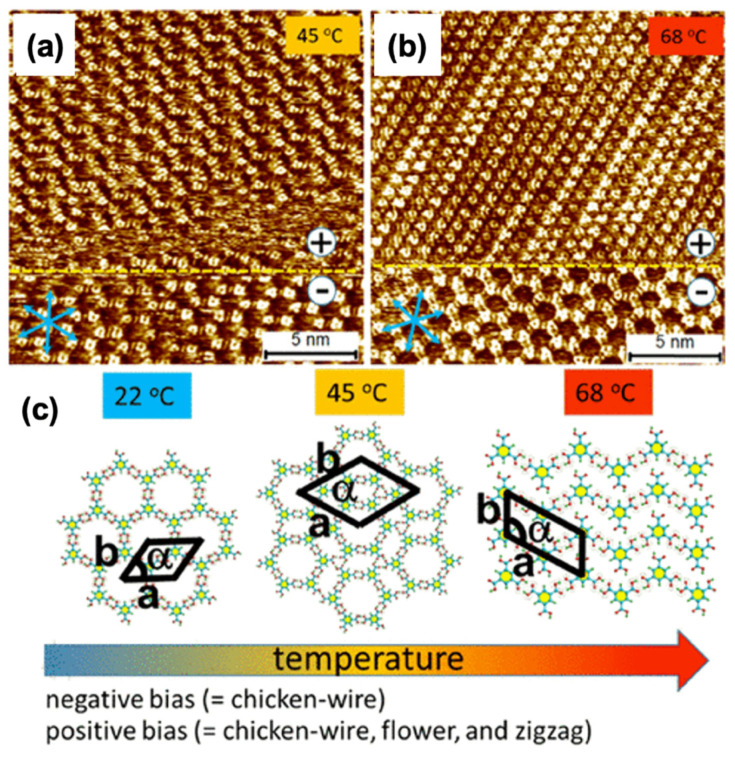
Represents temperature and electric polarity interrelated polymorphs showing the swapping between (**a**) chicken-wire, (**b**) flower and compact TMA assemblies. (**c**) Phase transformation diagram of TMA via thermal and electrical stimuli. Imaging conditions (V_bias,_ I_t_): ±0.85 V, 100 pA. The polarity of STM bias and the environmental temperature for STM imaging are noted in the image. Unit cell parameters a, b, and α for a chicken-wire motif for flower: 2.7 (±0.2) nm, 2.8 (±0.3) nm, and 60° (±2°); for compact: 1.0 (±0.2) nm, 1.8 (±0.2) nm, 87° (±3°). Blue arrows indicate the lattice-structure dimension of HOPG Reproduced from ref. [11] with permission from the American Chemical Society.

Furthermore, this finding also contradicts a few previous literature reports that a steady pattern of TMA porous networks might trap guest molecules, irrespective of the electrical polarity of the substrate [138]. Recently, Shern-Long Lee and his colleagues studied electric field-induced, including temperature-assisted supramolecular phase alterations using TMA as a model structure at the liquid/solid interface [139]. They experimentally show that at a positive bias of a substrate, TMA nanostructure experienced a phase transformation from a porous network (22 °C) to a flower structure (45 °C), and finally forms compact (68 °C) assembly with increasing temperature as shown in Figure 9a–c. The packing density for these assemblies slightly varies, i.e., chicken-wire (0.78 molecules/nm^2^), flower (0.98 molecules/nm^2^), and compact (1.13 molecules/nm^2^). This transformation in TMA occurs due to partial deprotonation in the carboxyl clusters. It is experimentally evident that the change occurs at a positive bias of substrate and higher temperatures. These molecular dynamics pave the way towards a monitored supramolecular phase swapping under the influence of electrical-thermal stimuli [139].

Usually, photoswitchable moieties comprise diarylethene, spiropyran, or azobenzene, which were covalently associated with a non-photoresponsive nanostructure to attain tunable supramolecular network via light [140]. The major drawback of chemical treatment is low synthetic efficiency and intricate cleansing process. Recently, amphiphilic aromatic dipeptide, diphenylalanine, has fascinated huge consideration due to its configuration, dimensions, and switchable self-assembly [141]. It possesses small molecular weight gelation performance in various unpolluted pure organic solvents. Principally, gels may be converted to thermodynamically stable phase (from gel to sol transition) or crystals (from gel to crystal development) via temperature or the solute concentration [142,143]. Currently, the considerable focus remained on stimuli receptive gel crystal alterations on the way to create distinct nano- or microstructures, including spheres, plates, cylinders, fibers, rods, or floras [144]. Many experiments were conducted to study reversible gel-sol switches activated via non-thermal stimulations; however, the light was rarely explored for phase transformation. A novel scheme of the light control mechanism of phase switchable (amino acid-based) supramolecular links opens wide applications, specifically in optoelectronics or biomedicines. The non-covalent induction of the photoswitchable segment permits alterable light modulation of a dipeptide (irresponsive to light; non-photosensitive) supramolecular network. In the presence of light, a photo-acid generator discharges a proton and facilitates the dipeptide dissociation, which results in sol formation. In the dark, gel regeneration takes place via entrapping a proton [145]. Using the energetically isothermal adjustable gel–sol alteration, renewable configurations can be developed as a result.

**Figure 10:**
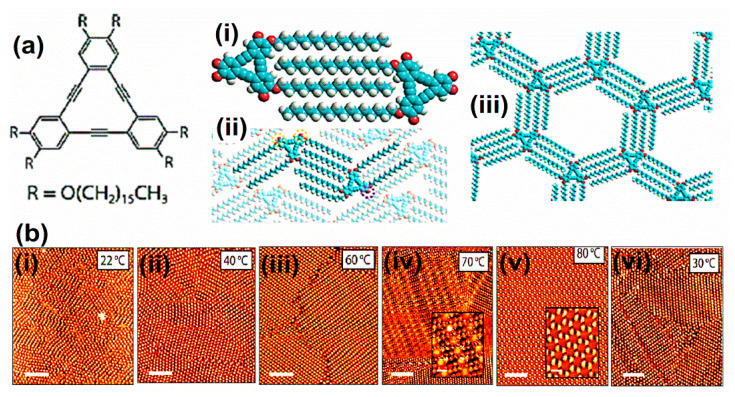
(**a**) Chemical structure of DBA-OC16. (**i**) The molecular model displays the interdigitation of alkoxy chains between adjacent DBA-OC16 molecules. (**ii**) Molecular model displaying the linear phase. (**iii**) Molecular model of the porous phase for DBA-OC16. (**b**) (**i**–**vi**) Thermal treatment on the transformation from linear-to-porous structure Reproduced from ref. [12] with permission from the American Chemical Society.

Steven De Feyter [146] and his group fellows studied the temperature-induced structural phase alterations in a 2D alkylated dehydrobenzo [12] annulene (DBA) self-assembled network. Their experiments demonstrate that the structural phase transitions occur via entropy deviations that form well-organized 2D molecular linkages. Figure 10 represents a compound configuration of DBA-OC16 and the associated molecular schemes displaying porous and linear phases. The molecular model displays the interdigitation of alkoxy chains between adjacent DBA-OC16 molecules. The model of the linear phase shows rows of tightly packed DBA-OC16 molecules. It is seen that the alternative rows of DBA molecules have four and five of their six alkoxy chains adsorbed on the surface. Four of the chains adopt a standard interdigitation configuration (Figure 10a), the fifth chain implements a bent configuration so that its length can lie parallel to other adsorbed chains. The molecular model of the porous phase for DBA-OC16 is shown in Figure 10b. Six alkoxy chains of DBA were adsorbed on the substrate; besides this, these chains are interdigitated with the adjacent DBA molecule chains developing a hexagonal nanopores system. Figure 10b displays STM results of successive annealing experimentation in which DBA-OC16 solution (5.8 × 10^−4^ mol/L) drop on HOPG at 22 °C, the temperature increment takes place in steps of 10 °C up to 80 °C, then cools to 30 °C. It is observed that at 22 °C; the composite possesses primarily the linear network with tiny domains of 5 to 20 nm having many packing flaws and domain boundaries (Figure 10b(i)). Annealing at 60 °C enhances the domain size and increases it from 50 up to 200 nm. At ~70 °C (Figure 10b(iv)), molecular rearrangement initiates as pores begin to appear, a porous phase, and a hexagonal pore generates within linear network domains. Figure 10b(iv,v) inset demonstrates transformation structures at ~70 °C and ~80 °C. At 80 °C, a complex system experiences widespread development to the porous phase. The subsequent assembly shows a well-arranged porous structure having domain boundaries appear at HOPG. The structure is cooled down in a lab environment to room temperature at 30 °C. At ~30 °C, it switches back to a linear arrangement.

Likewise, T. R. Linderoth explored a homochiral molecular nanostructure on the Au (111) substrate attained through chiral molecule co-adsorption, which acts as a switch with chiral induction seed [147]. Chen Wang [148] and his group fellows have explored the temperature-triggered chiral nanostructures. Before heating, achiral molecules self-assemble into strip morphology on HOPG. The R and S flower-like assemblies begin to generate at 55 and 60 °C annealing. The absorption energies estimated by DFT calculation demonstrate that R and S flower assemblies show more stability than strip assembly. S. De Feyter [149] efficaciously acquired enantiomorphous nanocavities with solvent-mediated chiral induction. The nanocavities might act as template complexes to HG assemblies on the way to establish chiral multi-component configuration. Moreover, L. J. Wan et al. have comprehended the hierarchical amplification of the homochirality of two-dimensional systems via the grouping of achiral molecules [150]. Thus, 2D chirality initiated by different external induced stimuli was also observed, leading to forthcoming practical instruments in chiral separation or chromatography. Previously, phase switches via an electric field on a gold substrate or stress-induced elongation were also performed in an atmospheric environment. These transformations were attributed to variations in the electronic surface excess charge density provoked via the electrical field between tip and substrate [151]. On Au (111) surface, the STM tip induces an electric field to cause reversible trans-cis isomerization of azobenzene derivatives [152]. Correspondingly, the transformation from a homogenous to homeotropic alignment occurs via induced electric field by trans-cis photoisomerization of azobenzene derivatives [153]. STM helps to eradicate Si atoms via negative sample bias (−3 V) depending on Si local bonding [154,155]. Likewise, Ni clusters deposit on SrTiO_3_ (001) were explored, displaying isolated clusters of varying sizes of the nuclei transformation towards bigger particles [156,157,158].

Additionally, the 2D H-bonded nanocomplexes also display special physiognomies via external influences, i.e., light radiation or thermal treatment. Henningsen and his colleagues studied the azobenzene derivative on Cu (1 0 0) substrate and elaborated its trans to cis isomerization [159]. Kumar debated alterable photoswitching in a single azobenzene [160]. The azobenzene-functional molecules can also be reversibly photoisomerized between trans- and cis-conformations through visible and UV light allocated to a molecule on- and off-state [160]. These kinds of light-controlled ordered metal-molecule-metal devices benefit future applications such as conductance switching, sensors, transistors [161,162], or as an anode material for energy storage applications in drug delivery emulsions.

## 11. Conclusions

At the beginning of HG chemistry in the 1980s, the essential principle of supramolecular chemistry briskly advanced due to the formation of diverse hosts, i.e., crown ethers, cryptands, pillararenes, or calixarenes. However, the investigation related to HG chemistry on compact substrates were initiated comparatively slow, perhaps due to the absence of appropriate modus operandi, which helps to access the hidden and out-of-the-way LS interface. STM specifically makes it possible to illustrate assemblies of intricate HG systems at submolecular resolution. These porous crystal complexes were exceedingly desired. Their elongated extended framework permits the elegant structural mechanisms required in molecular separation applications. As reviewed comprehensively above, HG interaction on compact substrates has progressed considerably in the preceding years. Initially, the host linkage structures were simple, in which the selection of guest atoms is usually limited to C_60_ or coronene. Various host’s structural design has been assembled on several solid planes to elaborate host void, strength, interactions, stability, or entrapping guest species. The development of efficient approaches in contemporary ages has revealed the immense potential to expand multifaceted and functional host arrangements. UHV–STM and LS interfaces have so far delivered illustrations of host–guest connecting systems, even though these are customarily qualitative consequences. The LS interface seems to be a methodology that can be scaled up for an upcoming device. The analyses conceded under UHV surroundings will prolong to have distinct prominence. The ultraclean environment with the nonexistence of solvent is a significant factor for straightening out important mechanical characteristics of guests associating progressions. Current development in the field specifies the mutual usage of the molecular scheme, supra-molecular amalgamation, and substrate interactions to comprehend HG arrangements designed for explicit purposes.

In recent times, novel approaches have been used to advance host systems that display guest-binding performance showing response to external stimuli. The research work reviewed currently discloses supramolecular chemistry concepts, which can be cost-effectively utilized in the ‘real-life’ approaches. In the real world, objectives of the host–guest binding investigated on solid planes can be advanced in two distinctive routes: its applications in molecular separations or sensing. The HG interaction on a substrate will be significantly improved via different essential approaches that permit the adjustment of a chiral molecule in 2D voids. This modification facilitates a careful HG group based on the compound and chiral molecule. A remarkable option is to consume the restricted area inside the nanocavities to operate the modifications. The dimensions of porous membrane voids which as a host may be altered according to the requirement to persuade the controlled reaction field. A well-defined restrain on the porous or dense intricate structure is an advantageous alternative characteristic for upcoming host structures. This procedure permit target molecules storage in open pores until, via using external stimuli, to close the arrangement. The examples deliberated above already own these desirable characteristics. However, there is a possibility for advanced investigation. Corresponding investigative methods such as emission spectroscopy or optical absorption will possibly be applicable to trace modifications in solution dilution on the release of guest molecules on the condition that guest release-capture takes place on an assessable balance. Moreover, in HG systems, idiosyncratic guests resist for adsorption might significantly advantage in Raman spectroscopy, i.e., receptive to the guest elemental analysis.

Besides these progressions, one cannot ignore the organic self-assembly kinetics and thermodynamics investigations at the LS interface, which is undergoing exciting and rapid succession. Through the last two epochs, the understanding of chemical reaction kinetics, i.e., rates of reaction or predicting the thermodynamic stability, is also debatable. In various chemical systems, the fundamental conflict takes place between thermodynamics and reaction kinetics in a system. It engenders significant arguments and comparisons between kinetics and thermodynamics. Consequently, at the LS interface, kinetic and thermodynamic mechanisms take place at LS to attain expecting surface assemblies associating with their chemical and nanoelectronic characteristics. Unluckily, quantitative knowledge of kinetics and thermodynamics is narrow compared to the qualitative explanation at the LS interface. The variables such as concentration, solvent, temperature, pressure, or time were considered external test center influencing parameters. However, at molecular dimensions, structure, polymorphism, the location of the process, i.e., boundaries, centers or defects, the interval at a specific position, or solvent co-adsorption, influence the supramolecular assembly. The desirable future aim is to investigate surface properties concerning different influencing variables and, by these means, govern entirely rate constants or thermodynamic variables linked together in progression. In the future, it is a prerequisite to consider the comprehensive information of solute solvation, solute–surface interaction, or occurrences of surface inhomogeneity due to defects, step edges, or reformations.

## Abbreviations

HG	Host–guest
LS	Liquid-solid
Sulflower	Termed after (1, C16S8), a planar D8h-symmetric octathio[8]circulene named from sulfur, and sunflower is the first fully heterocyclic circulene
ISA	Isophthalic acid
D	diameter
TA	Terephthalic acid
vdW	van der Waals
BTrB	A homologue of TMA was attained by inserting phenylethyne spacer between phenyl rings and carboxyl clusters
TCBPB	Has a biphenyl linker between the central phenyl ring and the carboxylic groups
DBA	Twelve-membered phenylene-ethynylene macrocycle called dehydrobenzo[12]annulene (DBA), substituted by six flexible alkoxy chains self-assembled to form hexagonal porous 2D molecular networks via van der Waals interactions between interdigitated alkyl chains as directional intermolecular linkages
NC-Phn-CN	Dicarbo nitrile polyphenylenes
SOF	Supramolecular organic frameworks
HBN	Hexagonal boron nitride
COR	Coronene
QPTC	Quaterphenyl tetracarboxylic acid
TPTC	P-terphenyl-3,5,300,500-tetracarboxylic acid
NG	Nanographene
DBA-OC10	2-decanol, alkoxyalted dehydrobenzo annulene (DBA) derivative, (DBAs with longer alkoxy chains)
CW	Clockwise
CCW	Counterclockwise
NN4A	Bis(3,5-diacidic)diazobenzene
TCDB	1,3,5-tris(10-carboxy-decyl oxy)-benzene
BDBA	Benzenediboronic acid
**I**_t_	Tunneling Current
H	Hydrogen
a	Achiral
c	Chiral
